# Effect of epinephrine on the absorption of lidocaine following application to the oral mucosa in rats

**DOI:** 10.1186/s12903-021-01691-0

**Published:** 2021-07-01

**Authors:** Rui Sasaki, Katsuhisa Sunada

**Affiliations:** Department of Dental Anesthesiology, School of Life Dentistry at Tokyo, The Nippon Dental University, 1-9-20 Fujimi, Chiyoda-ku, Tokyo, 102-8159 Japan

**Keywords:** Epinephrine, Topical anesthesia, Lidocaine, Oral mucosa

## Abstract

**Background:**

We investigated the role of epinephrine in prolonging the localization of lidocaine on the oral mucosa and inhibiting its absorption in the blood of rats.

**Methods:**

We used 7–8-week-old pathogen-free Wistar male rats (n = 128) for our study. We divided them into the control group administered with ^14^C-labeled lidocaine hydrochloride gel only and the study group administered with ^14^C-labeled lidocaine hydrochloride gel with epinephrine. The medications were administered in the palatal mucosa of the rats. The amount of mucosa, palatine bone, and serum lidocaine was measured by radioactivity using a liquid scintillation counter and was observed using autoradiograms.

**Results:**

Initially, there was no significant difference in the lidocaine levels between the lidocaine and lidocaine with epinephrine groups in the palatal mucosa (751.9 ± 133.8 vs. 669.8 ± 101.6 ng/mg [2 min]). After 4 min, the values were significantly lower in the lidocaine with epinephrine group (1040.0 ± 142.8 vs. 701.2 ± 109.0 ng/mg [20 min]). After 40 min, the lidocaine level became significantly higher in the lidocaine with epinephrine group (586.8 ± 112.4 vs. 1131.3 ± 155.2 ng/mg [40 min]). Similar results were observed in the palatine bone and serum.

**Conclusion:**

Epinephrine prolonged the localization of lidocaine applied to the mucosa and inhibited its absorption into the bloodstream of rats. Clinical studies are required to evaluate the use of epinephrine-containing topical anesthetics on the oral mucosa.

## Background

Dental topical anesthetics are not only used to numb the injection site but also for minor soft tissue surgeries [1, 2], extractions [3, 4], and suppression of the gag reflex [5]. Lidocaine is a widely used injectable local anesthetic that may also be used topically. However, the duration of lidocaine may be inadequate due to its short half-life. The oral soft tissues have a rich network of capillaries that causes rapid absorption of lidocaine after its application. Using large doses of lidocaine may result in systemic toxicity. The application of large doses of benzocaine, which is also widely used as a dental topical anesthetic, to the oral mucosa causes methemoglobinemia [6]. These side effects can be prevented by inhibiting local anesthetic absorption, which can be achieved by inducing the contraction of local blood vessels. Epinephrine, which has a powerful vasoconstrictive effect, is added to dental lidocaine injections for this purpose. However, as the mucosal permeability of epinephrine is low [7], its addition to topical anesthetics neither extends the duration of anesthetic action nor prevents its rapid absorption from the mucosa [7–10]. Dental topical anesthetics containing epinephrine are not currently used. In the past, tetracaine and cocaine with added epinephrine were widely used as topical anesthetics for the skin [11, 12] . A topical anesthetic containing lidocaine, tetracaine, and epinephrine was developed, and studies were conducted to determine its efficacy and safety [13, 14]. The efficacy of other cutaneous topical anesthetics has also been studied [15, 16], and the addition of epinephrine has been regarded as an effective method of obtaining a sufficient anesthetic effect [17]. Hence, it is worth reinvestigating the effect of epinephrine with topical anesthetics on the oral mucosal surface since no specific study has yet fully explored this effect. Intra-oral injections would be more comfortable and safer if epinephrine could increase the topical anesthetic effect. Capillaries running immediately beneath the mucosa, which lacks a cornified epithelium, are believed to be more susceptible to the effect of epinephrine than those under the skin. We hypothesized that epinephrine prolongs the anesthetic effect of topical lidocaine on the oral mucosa. The primary outcome of this study was the amount of lidocaine in the oral mucosa and in the serum of rats when topical anesthetic was used with added epinephrine.

## Methods

This study was approved by the Animal Experiment Committee of the Nippon Dental University School of Life Dentistry (approval No. 10-29) and was conducted in accordance with the guidelines laid down by ARRIVE (Animal Research: Reporting of in vivo Experiments).

### Animals

One hundred and twenty-eight 7–8-week-old pathogen-free Wistar male rats (Tokyo Laboratory Animals Science Co., Ltd., Tokyo, Japan) were used for this study. We divided the rats into two groups: the control group administered with lidocaine only, and the treatment group administered with lidocaine with epinephrine. Each group included 64 animals. Two rats per cage were housed in the animal room of the University’s isotope facility and were given unrestricted access to water and food.

### Formulation of test drugs

- Lidocaine

We dissolved 3.5 g of carboxymethyl cellulose sodium salt (CMC) in 100 mL of 2% lidocaine hydrochloride solution to formulate a 2% lidocaine hydrochloride gel.

Then, we added 25 µL of ^14^C-labeled 2% lidocaine hydrochloride (American Radiolabeled Chemicals, Inc., St. Louis, MO, USA) and 5 µL of 0.9% sodium chloride to 500 µL of 2% lidocaine hydrochloride gel.

(b) Lidocaine with epinephrine

We added 5 µL of 1 mg/mL epinephrine in place of 5 µL of 0.9% NaCl as used in (a).

Indigo carmine dye was added to both test drugs to aid visibility in order to excise the correct area of maxillary tissue.

### Lidocaine measurement

Lidocaine was measured according to the method described by Akimoto et al. [18].

- Sample collection

**Fig. 1:**
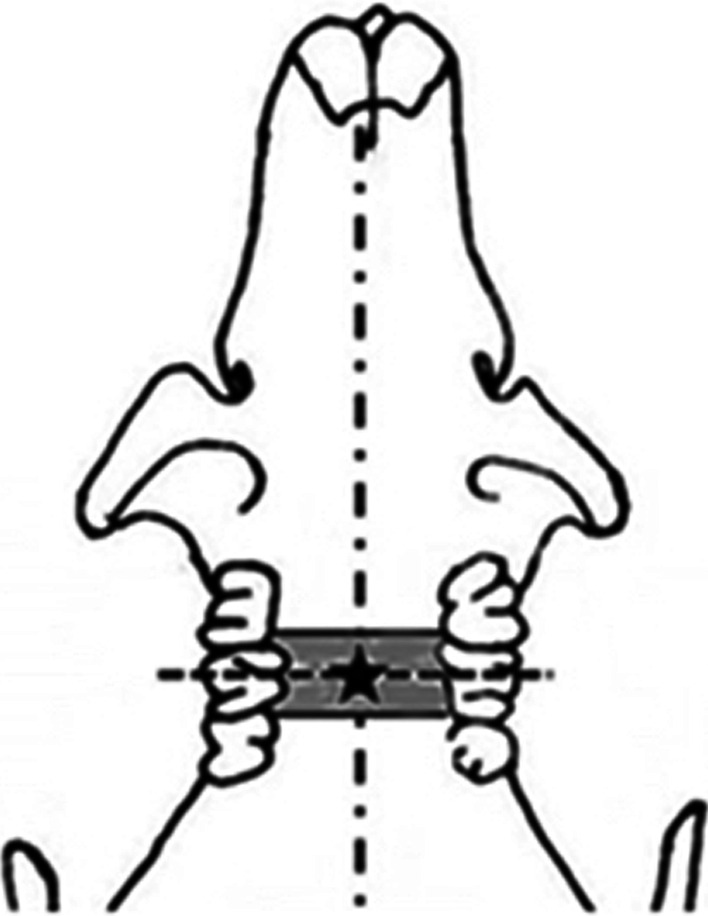
Application area of test drugs. Asterisk: indicate of application area. This point was the intersection of the midline of the palate and a line joining the centers of the bilateral second molars

Rats were administered pentobarbital (50 mg/kg) intraperitoneally for inducing sleep and were placed on their backs. A 5 µL dose of both the test drugs was applied to the oral mucosae of the animals of corresponding test groups using an applicator tip with an internal diameter of 2 mm and a length of 4 mm. This drug application was at the intersection of the midline of the palate and a line joining the centers of the bilateral second molars (Fig. 1). Samples of the maxilla and oral mucosa were collected from 100 rats, serum was collected from 24 rats, and autoradiographs were recorded for 4 rats.

(i) *Maxillary tissue*

One hundred rats (50 rats from each study group) were used for tissue measurement. A total of 10 time points were used in the study: 0.5, 2, 4, 7, 10, 20, 30, 40, 50, and 60 min after application. Sleeping rats were decapitated with a guillotine and the gel remaining on the surface of the mucosa was removed with cotton swabs. Thus, there were 5 samples at each time point. The drug-applied region was excised from the upper jaw using bone scissors, and the mucosa was separated from the bone using bone forceps. These samples of the mucosa and bone were minced with the bone scissors for radioactivity measurement.

(ii) *Serum*

Another 24 rats (12 rats from each study group) were used for serum evaluation. At 0.5, 2, 5, 10, 20, 30, 40, 50, and 60 min after application, 0.4 mL blood was collected from the left femoral artery and centrifuged at 4 °C and 15,000×*g* for 20 min to obtain the serum. Thus, 12 blood samples for each time point were collected from each rat of both groups. The study animals were euthanized by intraperitoneal administration of 150 mg/kg pentobarbital sodium after sample collection.

(b) Radioactivity measurements

The collected samples of the maxillary tissue (10–50 mg) or serum (50 µL) were placed in a liquid scintillation counter vial, and 0.5 mL of tissue solubilizer (Solvable®; PerkinElmer, Waltham, MA, USA) was added. This mixture was warmed and agitated at 60 °C for 2 h, and 25 µL of acetic acid was added to neutralize it.

To this solution, a liquid scintillation cocktail (AQUASOL-2®; PerkinElmer, Waltham, MA, USA) was added, and the resulting solution was left in the dark for 24 h. Thereafter, the radioactivity (dpm) was measured using a liquid scintillation counter (LEC-6100; Aloka, Tokyo, Japan).

The amount of lidocaine in the mucosa or palatine bone was calculated per wet weight of tissue (ng/mg wet weight) from the measured value and specific activity. The amount of lidocaine in the serum was indicated in terms of ^14^C-radioactivity (dpm/mL).

### Autoradiography observations

Autoradiography was conducted according to the method described by Hashimoto et al*.* [19].

- Section preparation

The remaining 4 rats (2 rats from each study group of lidocaine and lidocaine with epinephrine) were used for autoradiography. The same method of lidocaine measurement was followed as in the previous section. The maxilla was removed 10 and 40 min after lidocaine or lidocaine with epinephrine application, embedded in CMC, and placed on hexane dry ice to prepare frozen maxillary tissue blocks. A cryomicrotome (CM4050S®; Leica Microsystems, Wetzlar, Germany) was then used to prepare coronal sections of 10 µm thickness. These sections were placed on an adhesive sheet (Transfer Film®; Leica Microsystems, Wetzlar, Germany) and dried.

(b) Film observations

The dried sections were pressed onto an x-ray film (BioMax® XAR Film; Kodak, Rochester, NY, USA) and exposed to a temperature of − 80  C for 40 days. The developed films were placed on sections stained with 0.25% eosin (EosinY®; Nacalai Tesque Inc., Kyoto, Japan), and the radioactive isotope distribution was observed using a transmission scanner (GT9500®; EPSON, Nagano, Japan).

### Statistical analyses

Measurements are indicated as means ± standard deviations. Measurements at each time point were compared between the two groups using an unpaired t-test or Welch’s t-test if unequal variance was observed, with *p* < 0.05 regarded as significant. A software was used for statistical analyses (IBM SPSS® Statistics ver. 25; IBM Japan Ltd., Tokyo, Japan).

## Results

### Lidocaine measurements

- Palatal mucosa

There was no significant difference in the lidocaine levels in the first 2 min between the lidocaine group and the lidocaine with epinephrine groups. However, lidocaine values were significantly lower in the lidocaine with epinephrine group than in the other group at 4–20 min. After 30 min, this significant difference was no longer evident. However, after 40 min, the lidocaine level was significantly higher in the lidocaine with epinephrine group than in the other group (Table 1).

**Table 1:** Amount of ^14^C-lidocaine in the palatal mucosa

	Lidocaine Mean ± SD	Lidocaine with epinephrine Mean ± SD	*p* value
Sample size	5	5	
Time (min)			
0.5	371.5 ± 48.4	423.9 ± 71.2	0.210
2	751.9 ± 133.8	669.8 ± 101.6	0.306
4	813.5 ± 41.2	612.2 ± 56.2	< 0.001
7	948.3 ± 104.9	583.7 ± 47.5	< 0.001
10	1216.6 ± 156.7	658.5 ± 92.0	< 0.001
20	1040.0 ± 142.8	701.2 ± 109.0	0.003
30	940.1 ± 144.2	881.0 ± 84.7	0.452
40	586.8 ± 112.4	1131.3 ± 155.2	< 0.001
50	481.0 ± 53.2	995.2 ± 156.4	< 0.001
60	306.6 ± 109.0	621.5 ± 137.7	0.004

Data presented as the mean amount of lidocaine in ng/1 mg mucosa

*p* value compares lidocaine versus lidocaine with epinephrine

Unpaired t-test was used to compare means

*SD* standard deviation

(b) Palatine bone

There was no significant difference in the lidocaine levels in the first 4 min between the two groups. However, at 7–20 min, the lidocaine values were significantly lower in the lidocaine with epinephrine group than in the other group. From 30 min onwards, the lidocaine levels were significantly higher in the lidocaine with epinephrine group than in the other group (Table 2).

**Table 2:** Amount of ^14^C-lidocaine in the palatal bone

	Lidocaine Mean ± SD	Lidocaine with epinephrine Mean ± SD	*p* value
Sample size	5	5	
Time (min)			
0.5	1.8 ± 0.4	1.9 ± 0.5	0.738
2	3.9 ± 0.6	4.3 ± 0.6	0.378
4	3.1 ± 0.9	2.9 ± 0.6	0.769
7	5.2 ± 0.5	3.6 ± 0.9	0.011
10	10.3 ± 2.2	3.7 ± 0.6	0.002
20	7.4 ± 0.8	4.4 ± 0.6	< 0.001
30	4.3 ± 0.8	6.8 ± 0.6	0.001
40	3.4 ± 0.7	9.9 ± 1.2	< 0.001
50	2.6 ± 0.6	6.6 ± 0.6	< 0.001
60	1.7 ± 0.8	3.5 ± 0.8	0.008

Data presented as the mean amount of lidocaine in ng/1 mg bone

*p* value compares lidocaine versus lidocaine with epinephrine

Unpaired t-test or Welch’s test if unequal variance used to compare means

*SD* standard deviation

(iii) Serum

There was no significant difference in the lidocaine levels in the first 5 min between the lidocaine and lidocaine with epinephrine groups. However, after 5 min, and up to and inclusive of 20 min, the lidocaine levels were significantly lower in the lidocaine with epinephrine group than in the other group. After 30 min, this difference was no longer significant, although after 40 min, the lidocaine values were significantly higher in the lidocaine with epinephrine group (Table 3).

**Table 3:** Radioactivity of ^14^C-lidocaine in the serum

	Lidocaine Mean ± SD	Lidocaine with epinephrine Mean ± SD	*p* value
Sample size	12	12	
Time (min)			
0.5	19.1 ± 9.2	15.6 ± 6.7	0.291
2	21.8 ± 10.4	24.8 ± 5.6	0.383
5	34.1 ± 9.5	30.7 ± 6.6	0.322
10	55.9 ± 4.3	40.9 ± 4.8	< 0.001
20	76.9 ± 8.2	48.4 ± 8.6	< 0.001
30	63.4 ± 7.8	59.8 ± 4.7	0.189
40	49.3 ± 5.7	70.2 ± 4.4	< 0.001
50	34.8 ± 7.3	88.2 ± 2.9	< 0.001
60	20.6 ± 9.5	81.0 ± 4.8	< 0.001

Data presented as the mean of ^14^C radioactivity in dpm/1 mL serum

*p* value compares lidocaine versus lidocaine with epinephrine

Unpaired t-test used to compare means

*SD* standard deviation

### Film observations

**Fig. 2:**
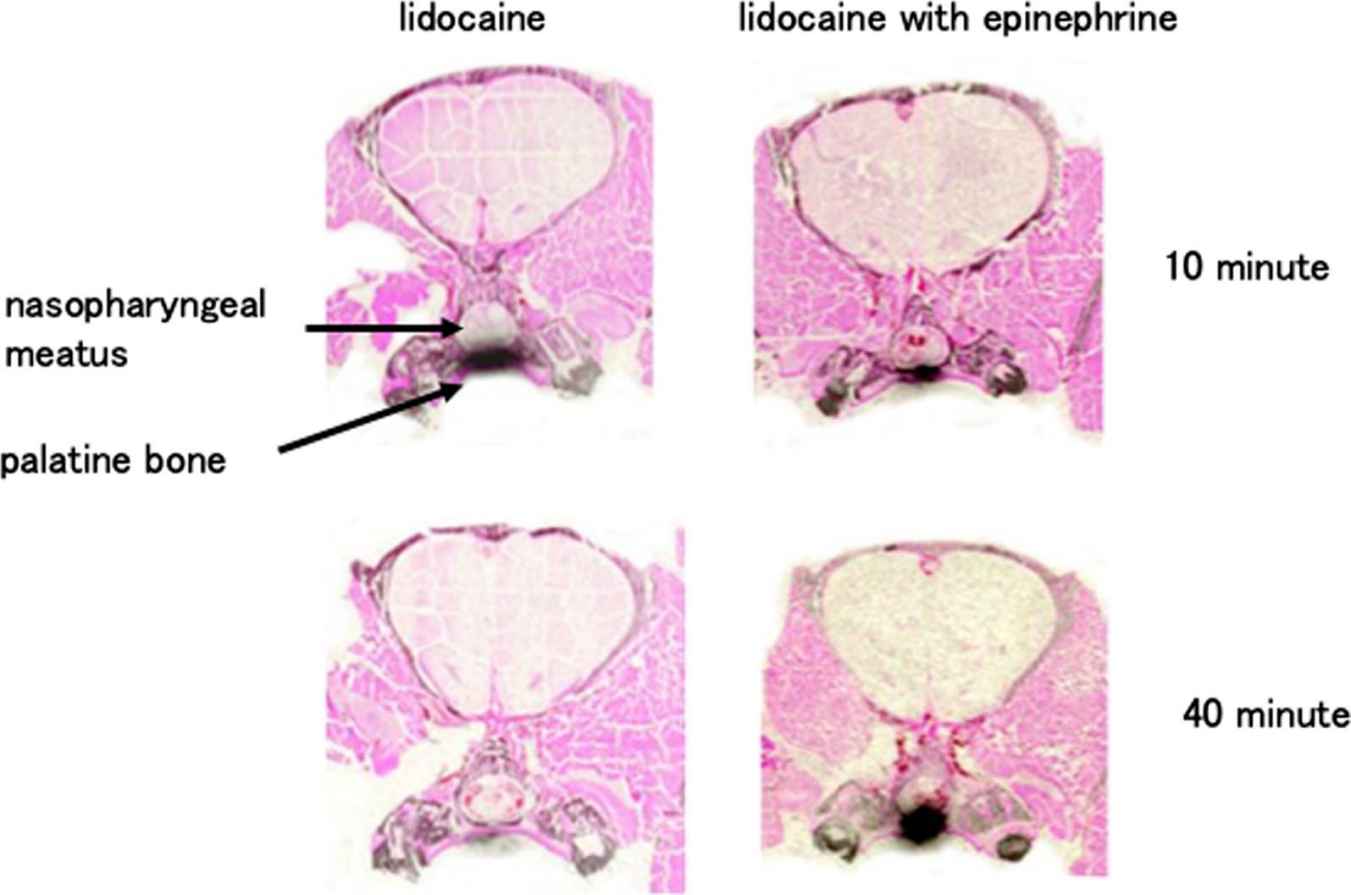
Autoradiograms of ^14^C-lidocaine in the coronary sections of maxilla. The maxilla removed at 10 and 40 min after the application of ^14^C-lidocaine (left) or ^14^C-lidocaine with epinephrine (left) was frozen, and a 10 μm frontal section was sliced at the center of the site of drug application. The blackened area shows accumulation of ^14^C-lidocaine. After 40 min, the blackened area increased in the maxillary section treated with lidocaine with epinephrine. In the maxillary section treated with lidocaine only, the blackened area almost disappeared

When epinephrine was added, the amount of radioactivity was lower after 10 min and higher after 40 min compared with that of lidocaine alone (Fig. 2).

## Discussion

The addition of epinephrine to lidocaine increased the time required for peak lidocaine concentration in the palatal mucosa and maxillary bone. The concentration of lidocaine in the mucosa peaked at around 10 min after application. When epinephrine was added, lidocaine concentration peaked at around 40 min. If the lidocaine concentration in the mucosa is correlated with its topical anesthetic effect, then the addition of epinephrine extends the duration of this effect.

Conversely, the slower rate of increase in lidocaine concentration in the mucosa may delay the onset of its effect as a topical anesthetic. However, studies have found that an adequate anesthetic effect is achieved within 1–3 min of its application to the gingival mucosa [20, 21]. In this study, the addition of epinephrine did not significantly affect lidocaine concentration up to 2 min after application. Therefore, we considered it unlikely that the addition of epinephrine delayed the onset of lidocaine’s topical anesthetic effect.

With the addition of epinephrine, lidocaine still reached the maxillary bone after 40 min of application. The extended time for which lidocaine was localized in the mucosa may have increased the amount that penetrated the maxillary bone. This suggests that clinicians must wait sufficiently after application of epinephrine-containing topical anesthetics before the initiation of invasive procedures, such as injection and deciduous tooth extraction.

As the oral mucosa is rich in capillaries, topical anesthetics are rapidly absorbed into the bloodstream. Although no studies have been performed on the human oral mucosa, when lidocaine is sprayed on the upper airway mucosa, the concentration in blood reportedly peaks after 5–30 min [22–25] . This rapid rise of lidocaine concentration in blood is more likely to cause toxicity [10, 26, 27]. In our study, the time required to reach the peak blood lidocaine concentration was increased from 20 to 50 min after application due to the addition of epinephrine. This indicated that epinephrine inhibits the absorption of lidocaine into the bloodstream, thus lowering the rate at which the blood concentration rises. However, the peak concentration in the blood was higher when epinephrine was added. These findings warrant further studies to investigate the effect of epinephrine on the risk of local anesthetic toxicity. It has also been reported that it may affect the synthesis of methemoglobin, which occurs as a result of the metabolism of local anesthetics [28].

Campbell et al*.* [10] and Adrian et al*.* [26] reported that the addition of epinephrine to the topical tetracaine applied to the mucosa of the pyriform fossa and trachea neither prolonged the duration of anesthesia nor inhibited their absorption. Several other studies have reported that the anesthetic action of lidocaine applied to the mucosal surface peaked after 2–5 min. Epinephrine had low tissue permeability, and its addition to topical anesthetics had no effect on its duration of action [7, 9, 26]. As epinephrine is strongly polarized in aqueous solution, it is unable to pass through the cell membrane [29]. Since the cell membrane is composed of a lipid bilayer, it is more easily penetrated by fat-soluble substances, and as epinephrine is a water-soluble hormone with a receptor on the cell membrane surface, it has low fat solubility. This is the reason for its low tissue permeability.

The reasons for the difference between these reports and our results are unknown. However, it is possible that epinephrine had no effect on the capillaries running through the deep mucosa when applied to the surface. It may have only constricted the vessels running immediately beneath the mucosa to prolong the localization of lidocaine. This suggests that it may not be necessary for epinephrine to permeate the deep mucosa to inhibit the absorption of topical anesthetics into the bloodstream. We also accurately measured the lidocaine concentration in tissues using radioisotopes, while all previous reports were clinical studies. It is possible that epinephrine’s effect may not be sufficient to affect the clinical action of anesthetics. As the addition of epinephrine delayed the increase in lidocaine concentration in the mucosa from 4 min after application, the possibility that previous studies may have measured its effect before the complete manifestation of its anesthetic action cannot be excluded.

This study has some limitations. We did not directly measure the anesthetic effect of lidocaine with epinephrine. We also did not investigate the suppression of toxicity or methemoglobinemia. Further clinical research to evaluate the effects of local anesthesia with epinephrine and animal studies to determine the decreasing effect for systemic toxicity of anesthetics and blood concentration of methemoglobin are required.

The results of this study demonstrated that epinephrine prolonged the localization of lidocaine applied to the oral mucosa and inhibited its absorption into the bloodstream. Therefore, further studies are required to investigate the clinical application of epinephrine-containing topical anesthetics on the oral mucosa. Use of epinephrine-containing topical anesthetics could make oral injections less painful and safer during dental procedures.

## Data Availability

The datasets generated during the current study are available in the Mendeley repository, Sunada K., Sasaki R. (2021). *Effect of Epinephrine on the Absorption of Lidocaine Following Application to the Oral Mucosa in Rats.* https://doi.org/10.17632/65pz7shjxy.1.
